# The Concept of the Optimal Bioscaffold: Parameters, Problems, and Their Resolution Through Additive Manufacturing

**DOI:** 10.3390/biomedicines13112688

**Published:** 2025-10-31

**Authors:** Petar Valchanov, Yordanka Yaneva, Stoyan Pavlov, Andreas Kontny, Tsanka Dikova

**Affiliations:** 1 Department of Anatomy and Cell Biology, Faculty of Medicine, Medical University of Varna, 9002 Varna, Bulgaria; yordanka.yaneva@mu-varna.bg (Y.Y.); stoyan.pavlov@mu-varna.bg (S.P.); andreas.kontny@mu-varna.bg (A.K.); 2 Department of Dental Materials Science and Prosthetic Dental Medicine, Faculty of Dental Medicine, Medical University of Varna, 9002 Varna, Bulgaria

**Keywords:** tissue engineering, scaffold, additive manufacturing, cell biology, 2D/3D model, in vitro model

## Abstract

In regenerative medicine, an engineered tissue is a composition of a sample of cells cultured on a spatially controlled medical device, called a biological scaffold (or just a bioscaffold). These devices are made of tissue-equivalent materials and represent the biological, mechanical, and spatial conditions in a specific type of human or animal tissue, becoming a possible way to replace damaged structures and develop artificial tissues or organs. Scaffolds with narrowly controlled characteristics—biological, mechanical and spatial properties—are vital for experiments mimicking in vivo conditions and in tissue regeneration scenarios. The aim of this narrative review is to identify and discuss the most important properties of these artificial constructs and the ways to achieve them via 3D printing-based technologies. Properties that can direct the development and differentiation of the cultured cells in a specific direction and ensure their biocompatibility and bioresorption, mechanical properties, spatial architecture, and porosity are discussed. The most common considerations in terms of the role of material selection, additives, and signal molecules and the appropriate spatially controlled manufacturing technologies for their assembly are covered, as are the radiological, biomechanical, and histological methods for their analysis. Finally, this paper highlights the challenges to the achievement of optimal scaffolds through additive manufacturing and gives suggestions for further research and development in this field.

## 1. Introduction

From ancient times to the present, one of the biggest dreams has been the replacement of damaged structures of the human body with artificial constructs to the point of a complete morphological and functional restoration. The design and manufacturing of engineered tissues with controlled geometry, mimicking the conditions of the extracellular matrix, is one of the major trends in Biomedicine in Industry 4.0. The main goal is to provide a unique environment for the culturing of specific cell populations and their morphological and functional integration. The conditions of those constructs depend on several factors with crucial importance for the survivability, development, and specialization of the cells in desired direction. These factors need to be considered in the earliest design stages, and the ability to precisely control them can provide new models for pharmacological and pathological experiments and new therapeutic approaches for regenerative medicine and implantology.

One of the concepts in tissue engineering is to combine biofactors (cells, proteins, growth factors, special additives, etc.) within an artificial, degradable porous construct with a controlled geometry, known as a scaffold. As Scott J Hollister said, “The art of scaffolding is where to put the holes and the biofactors” [1]. From a cell biology perspective, this means precise control over the properties and relationships in the entire scaffold–biofactor system. Biofactors can exert various functions, augmenting the general features of the scaffold and turning it into a complex, living, artificially engineered construct combining the positives of both worlds. A successful scaffold should attain a balance of structural, biological, and culturing factors, providing the cell biologist with precise instruments for control over its microenvironment, thus navigating the fate of the cells in the desired direction [2]. This paper reviews the most important parameters and how their proper integration can achieve the optimal scaffold.

From the perspective of biomedical and tissue engineering, a scaffold is a medical or scientific device that mimics both the morphology and the conditions of the native extracellular matrix of animal and human tissues and is manufactured with resorbable tissue-equivalent materials. Some scaffolds can be composed of unresorbable materials, which provide a stable, bioconductive environment for the ingrowth of the extracellular matrix into the device; examples include stent–graft prostheses [3] or metallic augments for bone substitution [4]. Most bioscaffolds are still personalized devices, and they are not considered medical devices according to the Medical Device Regulation (MDR2017-745) [5]. Instead, they are considered ATMPs (advanced therapy medicinal products). Criteria for ATMPs are set out in Article 17 of Regulation (EC) No 1394/2007 [6].

In the design and fabrication of an ideal (or at least optimal) scaffold, a number of conditions must be considered and precisely controlled.

A **biocompatible** [7] and **bioresorbable** scaffold [8] provides an environment suitable for the proper adhesion, proliferation, and differentiation of different cell types. Implantable scaffolds should additionally exhibit inflammatory and immunological inertness, thus avoiding any adverse reactions that may disrupt tissue stability and cause host rejection. The use of bioresorbable materials for fabrication ensures the substitution of the scaffold by a newly generated innate extracellular matrix and the successful organization and integration of the implant into the host. For a material to be considered biocompatible and bioresorbable, neither the material nor its degradation products should trigger adverse reactions or pathophysiological responses at any level of organization in the host. In fact, the concept of biocompatibility has changed over the years from “ability to not be rejected” to “the ability of a material to locally trigger and guide the proteins and cells of the host toward a non-fibrotic, vascularized reconstruction and functional tissue integration”, as defined by Crawford et al. in 2022 [9]. This means that the substance should degrade at a rate that matches tissue regeneration, preventing premature collapse or the prolonged persistence of foreign material. Therefore, choosing the appropriate biomaterials is critical. There are no ideal materials, and each type of polymer has advantages. Natural polymers like collagen, gelatin, and alginate offer excellent biocompatibility, while synthetic polymers like PLGA, PCL, and PEG allow better control of the degradation rates and mechanical properties [10]. Composite materials combining both types are increasingly used to balance bifunctionality with durability [11]. In non-natural materials, cell adhesion may be facilitated by the controlled addition of binding sequences to the substrate, such as RGD motifs [12].

The **crosslinking/gelation/polymerization mechanism** directly impacts compatibility as it may involve undesirable changes to the environment (temperature fluctuations, changes in ion concentrations and the strength of the extracellular fluid, the use of toxic catalysts, et cetera) or the generation of toxic by-products, thus triggering adverse reactions [13].

The scaffold architecture is an immediate result of the interaction between material properties, the gelation/polymerization mechanism, and the specific manufacturing process. From a biofunctionality perspective, a scaffold should be engineered to possess a tissue-specific porous structure, with a sufficient fraction of the total volume occupied by interconnected spaces. Optimal porosity ensures sufficient elimination of waste products, adequate cell migration, and the diffusion of nutrients [14].

In addition to its biocompatibility and biofunctionality, a **mechanically compatible** scaffold exhibits mechanical properties that vary within the physiological limits characteristics for the specific host tissues and preserved integrity from the moment of implantation until the completion of its organization and remodeling.

Manufacturing **technology** both depends on and influences all previously mentioned conditions and properties. An additional requirement is that the scaffold should be readily manufacturable with spatially controlled technologies that are scalable and ensure a high level of reproducibility (e.g., various additive 3D printing methods).

**Continuous and post-processing quality control** ensures adherence to standards and the production of an ATMP with the desired properties. During production, quality control measures should strictly observe parameters such as the pore size distribution, mechanical strength, degradation rate, and cytotoxicity. As manufacturing is a multistep process, an important additional requirement is to prevent the product’s contamination with pathogenic organisms. While measures for contamination reduction should be taken at every step, breaches are unavoidable. Given the high risks posed by such a contamination breach for both device failure and the host, decontamination procedures should be implemented in the post-processing of the ATMP. Based on the material used to produce the scaffold, various combinations of sterilization methods and/or antibiotic treatments must be considered. Decontamination/sterilization must preserve the integrity of both the scaffold and any incorporated bioactive agents.

As already mentioned, the aim of scaffold production is the successful implantation and integration of the scaffold into a highly complex living organism for either experimental or therapeutic intervention. This type of application implies the ability to perform reliable continuous monitoring and follow-up. Therefore, the generated scaffold should exhibit **radiological properties** that are appropriate for a reliable radiological analysis with X-ray and other imaging methods. This requires tissue-equivalent radiological properties that ensure proper visualization of the internal structure.

For histological and histopathological follow-up, the engineered matrix must possess appropriate **staining properties.** It should not interfere with the histological and cytological analytical techniques used for monitoring during scaffold development and follow-up examinations.

## 2. Biological Parameters of the Optimal Bioscaffold (The Roles of Bioscaffolds in Tissue Engineering)

Bioscaffolds are three-dimensional structures that support cell attachment, proliferation, and differentiation in tissue engineering and regenerative medicine. Acting as temporary matrices, these scaffolds mimic the natural extracellular matrix (ECM), guiding tissue formation by providing both mechanical support and biochemical cues that enable the development of cells into functional tissues [15]. A critical feature of these scaffolds is their ability to offer a surface for cell attachment, which is essential for tissue formation. Surface modification techniques, such as plasma treatment or coating with adhesive glycoproteins like fibronectin and laminin, can enhance cell–scaffold interactions, promoting more effective integration with the surrounding tissues [16,17].

Beyond providing attachment surfaces, scaffolds should facilitate cell proliferation and differentiation into the desired tissue type by incorporating growth factors, such as vascular endothelial growth factor (VEGF) and bone morphogenetic proteins (BMPs), that guide cellular development. Biocompatibility is a fundamental requirement for any scaffold in tissue engineering, ensuring that cells can attach, function properly, and migrate across the surface and within the scaffold. Furthermore, the scaffold must trigger a minimal immune response upon implantation to avoid inflammation that could hinder healing or lead to tissue rejection [18]. The long-term objective of tissue engineering is to allow the gradual organization and replacement of the implanted scaffold by the body’s cells and their newly produced ECM. Additionally, the degradation by-products must be non-toxic and efficiently eliminated from the body or naturally integrate into metabolism, avoiding disruptions to other organs and supporting (or at least not preventing) proper tissue regeneration [19]. Scaffolds must provide adequate mechanical support to maintain their structural integrity under physiological conditions while being sufficiently durable to withstand surgical handling during implantation. The choice of biomaterials and fabrication techniques, such as electrospinning and 3D printing, plays a significant role in determining the scaffold’s properties [20,21]. Biological tissues exhibit complex mechanical features such as heterogeneity, viscoelasticity and anisotropy, which pose challenges in accurately determining the mechanical properties required to design and manufacture scaffolds [22]. Ideally, scaffolds should possess mechanical properties corresponding to the specific anatomy of the implantation site. However, there is typically a balance between strength and porosity. Reduced porosity provides better strength, but potentially limits migration, metabolite diffusion, and vascularization. Therefore, finding a site-specific optimal balance between mechanical stability and porosity is important to support cell infiltration and blood vessel formation, which are both crucial for effective tissue regeneration [23].

The extracellular matrix (ECM) itself is a highly complex array of molecules that not only provides structural support to cells but also regulates their functions, behaviors, and interactions, modulating extracellular water equilibrium and regulating molecular diffusibility. The ECM exhibits spatial and temporal dynamics, with different tissues adapting its structure and composition to their specific needs. With recent advances in tissue engineering, the focus has shifted towards replicating the structure and function of the ECM with synthetic materials. This structural replication involves the recreation of important parameters of the ECM architecture, such as the isotropy of the structure [23], overall porosity (the percentage of empty spaces within the material) and minimal/maximal size of the pores [24]. Those parameters are crucial for the development of the cells in specific direction, and they can be manufactured through various materials, including nanomaterials, biomolecules, and synthetic polymers, offering new possibilities for scientific and therapeutic applications [25,26].

### 2.1. Diffusion Limitations

In scaffolds, like in native tissues, oxygen and nutrients are typically supplied by nearby blood vessels through diffusion, with a maximum diffusion distance under ideal conditions of up to 1 mm. Beyond this limit, passive diffusion cannot sustain adequate oxygen and nutrient delivery, leading to a rapid decrease in cell viability within the scaffold interior [27]. As a result, oxygen gradients frequently develop within scaffolds, and the oxygen concentrations are highest near the surfaces in contact with the culture medium and decrease significantly toward the core. Cells in areas with low oxygen levels may experience hypoxia, in turn leading to cellular dysfunction, degeneration, or death [28].

Consequently, for engineered tissues thicker than 1–2 mm, the incorporation of vascular-like structures or microchannel networks becomes essential to mimic the function of the native capillary bed [29]. Engineered conduits help to maintain a homogeneous oxygen and nutrient distribution, ensuring that no cell resides more than ~1 mm from an oxygen and nutrient source. Based on this diffusion constraint, a vascular or perfusion channel spacing of approximately 2 mm is generally required to sustain long-term cell survival and functional tissue organization within 3D constructs [30].

### 2.2. Hypoxia and Necrosis

Hypoxia activates various stress responses, including the upregulation of proinflammatory and apoptotic genes. Prolonged exposure to hypoxic conditions can lead to tissue necrosis, which is characterized by cell death, ultimately compromising the structural integrity of the scaffold. In addition to oxygen, cells rely on a continuous supply of essential nutrients, including glucose, amino acids, and other metabolic substrates, for their survival and proliferation. Insufficient diffusion within the scaffold can result in nutrient deprivation in certain regions, leading to cellular dysfunction or even cell death [31].

### 2.3. Factors Limiting Scaffold Functionality

In scaffold regions with an insufficient oxygen or nutrient supply, cellular proliferation and differentiation are diminished. This limitation decreases the overall effectiveness of the scaffold in replicating the functional characteristics of natural tissue.

The lack of oxygen and nutrient supplies impairs the formation of an organized, functional tissue. A well-functioning tissue requires appropriate cellular activity, including the proliferation and differentiation of cells, extracellular matrix (ECM) production, and cellular interactions. Limitations in diffusion can significantly impair these essential processes. Given this limitation, integrating a vasculature into bioprinted constructs is essential for ensuring the tissue’s continued growth and survival and maintaining normal physiological viability [32].

### 2.4. Strategies to Overcome Diffusion Limitations

Four valuable strategies have been designed to resolve the diffusion limitations of the bio scaffolds. They can be applied individually or in combination, significantly increasing the quality of the scaffold and the fate of the cells seeded on it.

- Promote vasculo- and angiogenesis within the scaffold via the incorporation of pro-angiogenic factors like VEGF (neovascularization) [33].
- Seeding of the scaffolds with endothelial cells in vitro to pre-form vascular-like structures prior to implantation, expediting the integration with the host’s circulatory system, and improving tissue survival (prevascularization) [34].
- Design and manufacturing of scaffolds with integrated microchannels or porous structures via microfabrication techniques that improve the internal distribution of oxygen and nutrients, thereby supporting cell viability throughout the construct [34].
- The incorporation of oxygen-carrying materials, such as perfluorocarbons, or the use of materials that facilitate oxygen transport, may provide additional means to address hypoxic conditions within the tissue, ensuring an adequate oxygen supply for cellular functions [35].

Better perfusion through dynamic environmental control within the bio scaffolds provides external control over the physicochemical environment by circulating a cell culture medium with consistent and well-defined parameters in the cell’s environment. This approach mimics blood circulation in the organism and helps to regulate important environmental properties such as pH, oxygen, glucose, and growth factors. It also facilitates the establishment of gradients for oxygen, growth factors, and other biochemical signals, supporting both cell–cell communication and interactions with the ECM [36]. Additionally, the customizable designs of microfluidic systems allow tailoring to specific tissue requirements, making them an effective tool for nutrient delivery in tissue engineering [37].

## 3. Manufacturing Technology and Material Selection

### 3.1. Materials Used for the 3D Printing of Bioscaffolds

According to materials science, there are three main types of materials (polymers, clays, and metals) [38], all of which can be candidates for the fabrication of scaffolds via additive manufacturing.

#### 3.1.1. Polymers

Polymers are the basic building blocks of living organisms and consist of multiple organic monomeric molecules (amino acids, nucleotides, carbohydrates, fats, etc.) connected into long fibrillar chains, forming the structures of the extracellular matrix and key parts of the cells in living tissue [39]. Together with the ground substance, they are an integral and mandatory part of any tissue, which makes them the perfect candidate as a tissue-equivalent material [40]. For tissue engineering applications, polymers can be used as rigid thermoplastics for biodegradable scaffolds [41], as water-based hydrogels for soft tissue scaffolds [42], or as specimen-derived decellularized extracellular matrix [43] as the ideal tissue-equivalent scaffold, both in terms of materials and structures. All of them provide a microenvironment similar to that of the natural ECM, which is crucial for cell survival and development under culture conditions.

**Thermoplastics**: Thermoplastics are a class of polymer materials defined by their ability to soften upon heating, enabling their moldability and extrudability in various fabrication techniques, including 3D printing [44]. They are frequently utilized in biotechnological applications due to their excellent mechanical properties, biocompatibility, and biodegradability [45]. They can be manufactured with filament, photopolymerization, or sintering-based 3D printing into a porous solid structure, which provides an adequate base for the development of multiple cell types [46]. Promising plastics for bioengineering applications are poly (α-hydroxy acids), including PGA, PLA, and their copolymer PLGA [47]. These polymers have a long history of use as degradable surgical sutures, and some of them have gained FDA approval for certain uses in humans and are reasonably biocompatible [48]. The ester bonds in these polymers are hydrolytically labile, and their degradation products are nontoxic, natural metabolites that are eliminated from the body in the form of carbon dioxide and water [49]. These polymers have already been used widely in bone tissue engineering research and there are ongoing research efforts to improve the functionality and further expand their applications. Other possible termopolymers include polyanhydrides [50], polycarbonates [51], polyaryletherketones [52], etc. Termopolymers can be further enhanced with additives like silver nanoparticles for antibacterial properties [53] or graphene for electroconductivity [54], making them even more versatile as scaffold building materials.

**Hydrogels**: Hydrogels are three-dimensional (3D) networks of polymer fibers dispersed in water [55]. They are based on polymers like alginate, methylcellulose [56], collagen [57], etc. The hydrogels in the gel state possess a specific viscosity that is low enough for direct extrusion 3D printing or sacrificial molding, and they can be further enhanced with additives like nanoclays [58], metallic nanoparticles [59], graphene [60], etc., to provide various additional properties. They can be solidified into a gel state through ionic, physical, photoinductive, or chemical gelation [13]. In the solid state, their internal composition of fibers mimics the natural extracellular matrix (ECM), providing a supportive environment for cell growth and proliferation. The presence of adhesive centers like laminin–fibronectin or RGD motifs provides good conditions for cellular adhesion, proliferation, and differentiation in the scaffold [26].

**Decellularized extracellular matrix (dECM)**: dECM is a promising bioengineering material that can be classified in two groups: tissue- or organ-derived ECM and ECM formed by cultured cells [60]. dECM is typically obtained through a combination of mechanical, chemical, and biological methods [61] that aim to eliminate cellular debris, including DNA and RNA, while retaining the essential ECM components such as collagen, glycosaminoglycans, and elastin, which are critical for cell adhesion, migration, and proliferation under culture conditions [62]. Such scaffolds are especially suitable for wound healing [63], vascular grafts [64], and parenchymal organs such as the liver or myocardium [62]. dECM scaffolds have several advantages over the hydrogel and thermoplastic scaffolds, including their low cytotoxicity and immunogenicity, which significantly increase their acceptance by host tissues compared to synthetic scaffolds [65]; this property is crucial for applications such as long-term tissue integration and function. The preservation of the native structural integrity during decellularization results in scaffolds with mechanical properties that can withstand physiological loads, an essential consideration for load-bearing tissues such as cartilage and bone [66]. Recently, there has been a significant development in the additive manufacturing of dECM scaffolds with a detailed and fine microarchitecture and high structural complexity, including cardiac tissue [67], blood vessels [68] or even pancreas [69], which makes it one of the most promising materials for tissue bioprinting.

#### 3.1.2. Metals

Metals are solid materials that are typically hard, shiny, malleable, fusible, and ductile, with good electrical and thermal conductivity, but some of them (titanium [70], tantalum [4], etc.) have high biocompatibility and osteoconductivity. They can be used to produce strong, highly porous, biologically inert constructs, acting as a strong foundation for the development of new tissue in the vacant spaces of the scaffold. The resulting metal–tissue arrangement provides excellent conditions for the development of a newly generated extracellular matrix that anchors the metallic object to the organic tissues [71]. Tantalum augments, called the trabecular metal system, have multiple applications in orthopedic surgery, including the augmentation of the acetabulum for revision surgery of the hip [72]. As stent grafts, metallic scaffolds are commonly used in endovascular aortic aneurysm repair (EVAR) operations [73]. Additionally, a new class of metallic biodegradable temporary stents have been developed whose presence is limited to a period of 6–12 months after implantation, during which arterial remodeling and healing occur. They are made of iron or magnesium alloys and offer promising new opportunities in the treatment of cardiovascular conditions [74].

#### 3.1.3. Clays

Clays are a mineral-based class of materials and one of the most promising candidates for osteogenic applications since minerals (like hydroxyapatite crystals) are an integral part of the bone tissue ECM [74]. They can be manufactured as pure ceramic scaffolds, such as hydroxyapatite [75] and calcium carbonate [76]; as a component in composites with termopolymers, such as polycaprolactone [77] and polylactic acid [78]; or as an additive in hydrogels, such as alginate/methylcellulose [42] or chitin [79] composites. Clays can be manufactured into scaffolds containing interconnecting spaces with optimal target porosity for the development and regeneration of osseous ECM and tissue-specific microcirculation, which makes them an excellent material for bone tissue engineering [80].

### 3.2. Manufacturing Technologies for the Fabrication of Scaffolds with Controlled Porosity

#### 3.2.1. Design Strategies for Manufacturing Bio Scaffolds with Controlled Porosity

In scaffold design, controlled porosity plays a critical role in tissue engineering, as it influences the diffusion of oxygen, nutrients, and waste products, mimicking the natural extracellular matrix (ECM) structure. To create a viable environment for cells to grow, proliferate, and form functional tissues, the design of scaffolds with the right porosity and interconnectivity is essential [81]. The microcirculatory system in tissues consists of small blood vessels that enable efficient nutrient and oxygen distribution to cells. In tissue engineering, this functionality can be reproduced by designing scaffolds with internal interconnected spaces that facilitate the optimal distribution of the culture media inside the matrix and its contact with the cells incorporated in the device. Interconnected pores within the scaffold increase the diffusion of the culture media and allow for a better distribution of oxygen, nutrients, and metabolites. This improved diffusion is particularly important for cells deep in the scaffold, overcoming the limitations imposed by the diffusion distance [82]. Additionally, these interconnected pores facilitate cell migration, which aids in tissue integration and the de novo formation of vascular-like networks. This process, known as angiogenesis, is critical and ensures long-term tissue survival [83].

Optimizing the porosity and channel geometry of scaffolds is essential for the creation of a tissue growth-promoting environment. The overall porosity, defined by the percentage of empty spaces within the material, plays a crucial role in regulating nutrient and gas diffusion. Typically, an ideal porosity ranges from 70% to 90%, depending on the specific tissue being engineered. However, excessive porosity can compromise the mechanical strength of the scaffold, while a porosity that is too low may limit cell growth [24]. Several techniques are employed to control porosity, such as salt leaching, where water-soluble salts are mixed with a polymer matrix to create a porous structure [84], and freeze-drying, which uses vacuum conditions to sublimate the solvent and leave behind a porous scaffold [85]. Additionally, advanced 3D printing technologies provide precise control over the pore size and geometry, allowing scaffold customization for specific tissue types [24].

The design of the channel geometry is just as crucial as porosity. Channels need to be appropriately sized and shaped to mimic the natural vascular network of tissues. When designing scaffolds for tissue engineering, optimizing the channel geometry is crucial for ensuring efficient nutrient diffusion and supporting cellular infiltration. The size and shape of the channels are important factors to consider; typically, channels should range from 50 to 500 microns in diameter [86]. If the channel diameter is too small, nutrient flow may be restricted, while excessively large channels could compromise the scaffold’s mechanical stability. The cross-sectional shape of the channels must also be tailored to mimic natural vascular networks, with cylindrical or branched channels being common choices. These shapes help exchange nutrients and waste products and support the formation of vascular-like structures within the scaffold [87]. Branching and alignment of the channels further enhance tissue mimicry by replicating the vascular tree structure found in natural tissues. Channel branching increases the surface area for nutrient exchange and supports angiogenesis. The alignment of the channels can be adjusted to mirror the anisotropic nature of the microcirculation in specific tissues, such as bone, muscle, or nerve tissue, where cells and vessels have a specific non-random orientation. This alignment promotes the natural organization and function of the tissue being engineered [88].

The choice of scaffold materials plays a critical role in controlling porosity and channel geometry. Materials such as hydrogels, bioceramics, and synthetic polymers can be engineered with a specific pore size and geometry. Biodegradable materials are often preferred because they provide temporary support while being gradually replaced by the growing tissue, ensuring that the scaffold degrades as the tissue forms and matures [89].

Beyond porosity and the channel geometry, the surface properties of the scaffold are also essential for promoting cellular interactions. Surface modifications, such as using ionized gases (plasma treatment) [16] or coating with adhesive glycoproteins like fibronectin or collagen [90], can enhance cell–scaffold interactions, promote more effective cell adhesion and differentiation, and guide cells to form organized tissues within the scaffold. These modifications can significantly improve tissue formation by promoting specific cellular behaviors [91]. Additionally, in some cases, the strength and mechanical performance of the scaffold can be significantly improved by incorporating nanoparticles and/or coatings, such as the usage of nanodiamond, hydroxyapatite, bioactive glass particles, SiO_2_, MgO, and silver nanoparticles [92].

Microfluidic systems represent an innovative approach to regulate porosity and increase the nutrient distribution within scaffolds. By incorporating microfluidic channels that replicate blood vessels, these systems create a controlled environment for tissue culture. The precise management of fluid flow within these channels improves nutrient delivery, waste removal, and overall cell behavior, thus promoting better tissue growth and functionality [32].

#### 3.2.2. Manufacturing Strategies for Scaffold Fabrication: Direct Fabrication vs. Sacrificial Molding

There are two main strategies used to manufacture a porous scaffold: direct manufacturing through the deposition of the material [93] or indirect manufacturing through molding with a sacrificial material [94].

Direct fabrication is the most common method. It builds the structures of the construct through the direct deposition of material only in the necessary locations and leaving the porous spaces empty. It is a simple, quick, and straightforward method that can be performed easily with any spatially controlled manufacturing technology, but it lacks the details and accuracy needed for the proper design of the scaffold.

The indirect fabrication method is more complicated and requires better planning, a more sophisticated setup, and more time for its proper execution. First, the structures of the empty spaces in the scaffold are built with a special sacrificial material. Then, the model of the empty spaces is used as a template, around which the material for the final product is cast. Usually, it includes hydrogel or clay in liquid form that solidifies at a later stage. After the solidification of the final material, the structures of the template are eliminated through dissolution with a solvent [95] or burning in a sintering furnace [96]. The resulting scaffold has a much more intricate and accurate internal structure with significantly improved quality and functionality of the construct.

#### 3.2.3. Additive Manufacturing Techniques for Scaffold Fabrication

**Figure 1:**
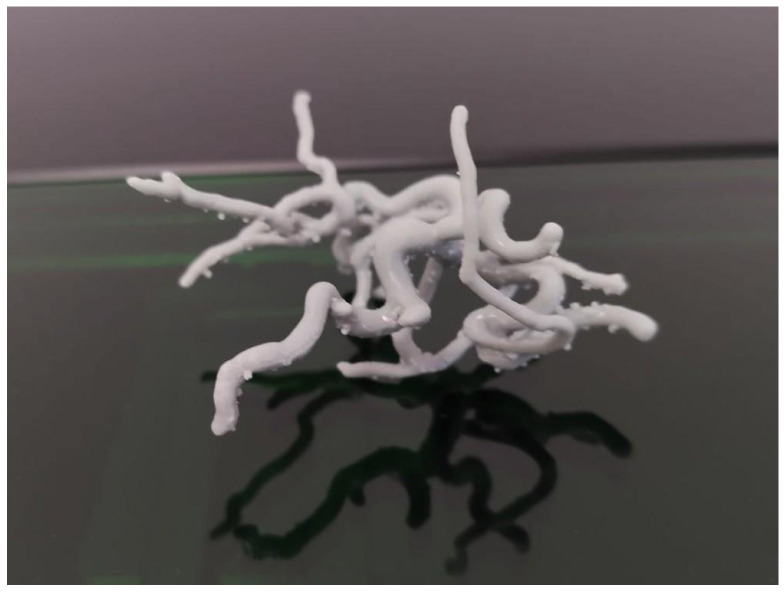
Three-dimensional printed model of a microcirculatory network.

**Stereolithography (SLA, DLP, and LCD)**: A polymer resin is deposited in a vat and polymerized layer by layer into a solid object by a source of light, such as a laser and visible or UV light display. This technology is capable of producing a layer thickness of 50–100 μm [97] and highly accurate complex geometries in a scaffold or a template for scaffold molding (Figure 1). Biopolymers like gelatin, collagen, and elastin can be functionalized with photosensitive groups and used similarly to photopolymer resins to manufacture objects with excellent biological properties tailored toward cell adhesion and viability. The downside of this technology is the high price of the materials and the need for support scaffolds for the hanging parts of the 3D printed object, which can compromise its structural stability.

**Selective Laser Sintering (SLS, SLM)**: This high-end additive manufacturing technology uses a high-power laser to fuse powdered material layer by layer, creating a physical object with high accuracy. This technology is self-supporting, because the unfused powder itself acts as a support structure, making this technology ideal for manufacturing strong, complex objects such as bio scaffolds [98]. The powdered materials with great promise for osteogenic applications include polymer–clay composites such as poly (caprolactone)/hydroxyapatite [99] and poly (vinyl alcohol)/hydroxyapatite [100], polymer/metal composites such as poly (l-lactic acid)/Mg [101], and bioceramics such as microhydroxyapatite/nanosilicate [102]. A serious limitation of this technology is the high sintering temperature (above 1200 °C), which makes the manufacturing of biological molecules impossible [103].

**Fused deposition modeling (FDM):** A thermoplastic filament is melted, extruded through a nozzle, and deposited on a building platform by a 3D printer, mostly an XYZ or CoreXY system. It is the only technology that can 3D print bridges in thin air between the supported parts of the scaffold, thus enabling the manufacturing of parts with a complex geometry and numerous unsupported areas. A broad range of thermoplastics and elastomers are available for FDM, which can be used with single or multiple materials; it is capable of producing a layer thickness of 100 μm, but it lacks the precision of stereolithography [104]. An important limitation of FDM for scaffold fabrication is the inability to 3D print cells with biological factors because of the direct effects of heat and pressure on them, although some interesting results have been obtained with pre-incubator bioprinters equipped with a standard FDM 3D printing nozzle [105].

**Figure 2:**
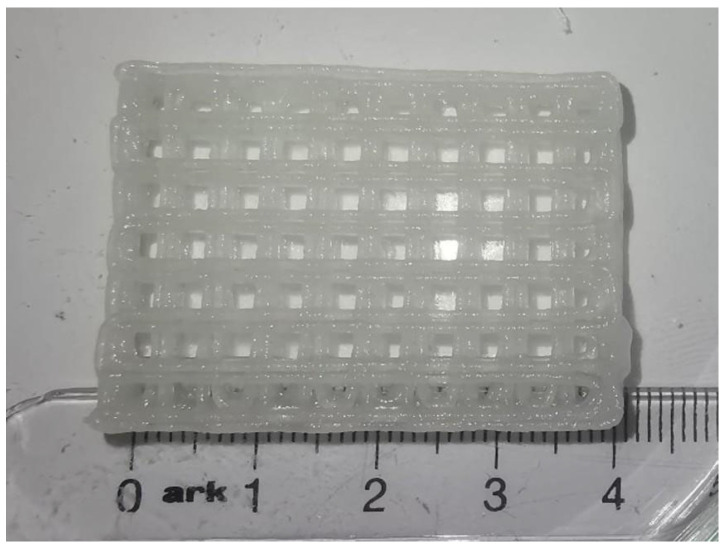
A scaffold that was 3D printed with a composite hydrogel containing alginate, methylcellulose and laponite using a Hyrel 3D Hydra 17A 3D printer with a syringe extruder [109].

**Direct extrusion 3D printing:** A bioink containing polymers is extruded through a syringe, air compressor, or piezoelectric system, deposited layer by layer on a build platform, and polymerized through ionic, physical, photochemical, etc., mechanisms [106]. Depending on the extrusion system, it can achieve a layer thickness down to 150–300 μm (Figure 2) and is capable of printing bridges in the space between the supported areas of the scaffold [107]. The scaffold can be sterilized before printing and manufactured under sterile conditions or after fabrication using traditional sterilization methods, and sterilization can also be considered a parameter that affects a construct’s resolution, structural integrity, and geometry [108].

A variant of the direct extrusion 3D printing is Freeform Reversible Embedding of Suspended Hydrogels (FRESH), where the hydrogel is extruded within a secondary semiliquid hydrogel that serves as a temporary, thermoreversible, and biocompatible support. After the completion of additive manufacturing, the secondary hydrogel is removed, enabling the 3D printing of complex objects with a resolution of ~200 μm [110].

**Inkjet 3D printing** is a droplet-based additive manufacturing method that is capable of producing complex three-dimensional structures with multiple materials, including polymers like poly (caprolactone) (PCL), poly (lactic acid) (PLA), and poly (lactic-co-glycolic acid) (PLGA) [111], or clays like alumina ceramic [112]. Although the dimensional accuracy of the inkjet 3D printing [113] is not as good as other methods like SLA [114], the advantage of inkjet bioprinting is that cells and other biofactors can be added to the printed material, improving bone induction and bone conduction of the scaffold [115].

**Comparison of the 3D printing methods for scaffold manufacturing:** Important questions for every 3D printing technology are its availability and cost. In this regard, extrusion-based 3D printing and stereolithographic methods like SLA or DLP are more affordable (with a reasonable resolution down to 100–200 μm), with a broad range of available materials and years of experience, which make them excellent candidates for bioscaffold fabrication. There are more accurate methods for 3D printing of objects with complex geometries, such as selective laser sintering, two-photon polymerization of photopolymers [116], or super-resolution electrohydrodynamic (EHD) 3D printing with phase-change inks [117] at micron or submicron resolution. A comparison of the advantages and disadvantages of the 3D printing methods is shown in Table 1.

#### 3.2.4. Current Gaps in Scaffold Fabrication

One of the most significant challenges in scaffold fabrication is scalability. While techniques that work effectively in small, laboratory-scale experiments often fail to maintain consistency and efficiency when scaled up to industrial production levels; this presents a major obstacle. Ensuring uniformity and cost-effectiveness at a larger scale are crucial for making scaffolds available for clinical applications, where these factors are of utmost importance [118].

Another challenge is the precision of the control of porosity in scaffolds. The porosity of a scaffold is essential for its functionality, as it facilitates cell migration, nutrient diffusion, and waste removal. However, achieving precise control over porosity remains difficult, as most current fabrication methods struggle to consistently replicate the desired pore size, shape, and distribution. This inconsistency can hinder the scaffold’s performance, and it becomes even more complicated when considering the specific needs of different tissue types, such as bone, skin, or cartilage, each requiring unique porosity configurations [24].

#### 3.2.5. Emerging Trends in Scaffold Fabrication

A promising trend in scaffold fabrication is the development of biofunctionalized scaffolds. These scaffolds are chemically modified to incorporate biological molecules, such as growth factors or extracellular matrix proteins, which can enhance cell attachment, growth, and differentiation. By improving the interaction between the scaffold and the cells, biofunctionalized scaffolds can guide the formation of specific tissues, significantly increasing the success rates of tissue regeneration. However, finding the right balance between biofunctionalization and maintaining the structural integrity of the scaffold remains a challenging task [119].

The integration of real-time monitoring technologies is another emerging trend in scaffold fabrication. Three-dimensional bioprinting has shown great potential in fabricating biological tissues by precisely depositing cells and bioinks. However, ensuring the quality and viability of these bioprinted structures remains a significant challenge. One solution being explored is the incorporation of sensors into scaffolds, allowing for continuous monitoring of environmental parameters such as pH, temperature, oxygen levels, and mechanical stress. Real-time data collection facilitates optimal tissue growth by providing a better-controlled microenvironment, offering the potential to improve experimental and clinical outcomes by enabling more precise, patient-specific therapeutic strategies [120].

An interesting trend in scaffold manufacturing is the synergistic use of 3D printing and electrospinning, overcoming their disadvantages—the low resolution of 3D printing and spatially uncontrollable electrospun fabrication—that limit their applications and performance [121]. It combines the advanced spatial control of 3D printing with nanofiber-incorporated bioinks and allows the fabrication of a new class of nanomaterials with advanced structural properties. Scaffolds can be fabricated with such materials to create a porous structure with pore size of 100–300 μm and porosity as high as 79%, which show great promise for osteogenic applications [122].

Interdisciplinary collaboration also plays a crucial role in advancing the field of scaffold fabrication. By bringing together expertise from biology, materials science, engineering, and clinical medicine, interdisciplinary teams are essential in developing bioactive materials and scalable manufacturing processes, and ensuring regulatory compliance. Additionally, collaborative research is vital for integrating emerging technologies, such as 3D printing, nanotechnology, and AI-driven design, to drive innovation in scaffold creation and improve therapeutic applications [123].

#### 3.2.6. Recent Developments: In Situ Bioprinting

In situ bioprinting is a recently developed method that enables the direct deposition of bioink (containing growth factors and/ or living cells) onto tissue defects in the living animal or patient. Unlike in vitro bioprinting, where scaffolds are printed externally and are implanted later, in situ bioprinting aims for direct integration into the wound. Bioink can be deposited with handheld (manual) devices, robotic arms, or even conventional 3D printers. Computer-controlled 3D printers offer higher precision than handheld devices but need a proper CAD model for path computation in advance [124].

Further research needs to be performed to determine if possibly longer surgeries for larger tissue defects treated with in situ bioprinting will be beneficial compared to the in-depth preoperative planning and in vitro production of scaffolds.

#### 3.2.7. Artificial Intelligence (AI) and Machine Learning (ML): Quality Control of Bioprinted Scaffolds

Recent advances demonstrate that artificial intelligence and machine learning can play a transformative role in improving quality control and process optimization in bioprinting. By analyzing large datasets of printing parameters, material properties, and biological outcomes, AI models can identify complex, non-linear relationships that are difficult to capture through conventional statistical methods. As illustrated by recent work in the field, data-driven models can accurately predict the structural and functional quality of printed scaffolds based on input variables such as the bioink composition, cell type, and printing conditions. These predictive frameworks allow researchers to virtually screen printing scenarios before fabrication, reducing the reliance on costly trial-and-error experimentation [125].

## 4. Conclusions

In conclusion, scaffolds play a crucial role in tissue engineering by replicating the extracellular matrix (ECM) and supporting cell growth and differentiation. Despite advances in scaffold design using 3D printing and other techniques, challenges remain, particularly in ensuring efficient nutrient and oxygen diffusion. Scaffolds must balance mechanical strength, biocompatibility, and biodegradability to promote tissue growth and vascularization. They can be readily manufactured with highly available technologies, such as FDM and extrusion-based or stereolithography-based 3D printing, and with reasonable quality for biomedical applications.

## Figures and Tables

**Table 1:** Comparison of the advantages and disadvantages of 3D printing technologies.

3D Printing Technology	Material	Advantage	Disadvantage
FDM	Termopolymers	Low cost Low toxicity	Support is required for overhanged structures Inability to print with cells and biomolecules Limited to Thermo polymers Medium quality
Inkjet	Various materials Biological materials	Can print with cells and biofactors	Low quality High cost
Direct extrusion	Hydrogels Biological materials	Can print with cells and biofactors Good for soft tissues	Low quality Support is required for overhanging structures The size of the nozzle limits it
SLA	Resin	Great quality Great fabrication speed	Biomaterials must be photo polymeric Support is required for overhanging structures High cost
SLS	Powders	Great quality Self-supporting technology Fabrication of scaffolds with good mechanical strength	High working temperature above 1200 °C High cost.

## Data Availability

Data are contained within the article.
